# Exosomal microRNA signatures in youth at clinical high risk for bipolar disorder

**DOI:** 10.3389/fpsyt.2025.1589374

**Published:** 2025-05-20

**Authors:** Xinyu Meng, Shengmin Zhang, Yingzhen Xu, Zaohui Ma, Shuzhe Zhou, Yantao Ma, Hong Ma, Xin Yu, Lili Guan

**Affiliations:** Peking University Sixth Hospital, Peking University Institute of Mental Health, National Health Commission (NHC) Key Laboratory of Mental Health (Peking University), National Clinical Research Center for Mental Disorders (Peking University Sixth Hospital), Beijing, China

**Keywords:** bipolar disorder, clinical high risk, miRNA, exosome, biomarker

## Abstract

**Introduction:**

Individuals at clinical high risk for bipolar disorder (CHR-BD) experienced insufficient recognition. Little is known regarding the association between exosome microRNA (miRNA) profile and bipolar disorder (BD) risk.

**Materials and methods:**

Twenty youth at CHR-BD, 21 patients with BD, and 24 healthy controls were recruited in this study. Exosomal small RNA sequencing was undertaken in the plasma sample of the participants. Using machine-learning algorithms, target miRNAs were selected from differentially expressed candidates. Predictive models were built and tested on validation set.

**Results:**

The study identified two miRNAs that showed significantly differential expression between the CHR-BD group and the HC group: hsa-miR-184 (log_2_FC = 4.22, *P* = 1.49E-04) and hsa-miR-196a-5p (log_2_FC = 4.75, *P* = 3.56E-04). Random forest (RF) and eXtreme Gradient XGBoost jointly selected two overlapping miRNAs: hsa-miR-1908-3p and hsa-miR-412-5p. XGBoost outperformed the RF model with higher AUCs (BD group: 0.71 vs 0.71, CHR-BD group: 0.74 vs 0.72, HC group: 0.60 vs 0.57).

**Conclusion:**

The study identified four target miRNAs involved in neuroimmunity and neuronal plasticity, supported by literature linking these miRNAs to neuropsychiatric diseases, suggesting their potential as biomarkers for early BD. Future research should integrate additional biomarkers for improved discriminative performance.

## 1 Introduction

Bipolar disorder (BD), characterized by recurrent episodes of mania (hypomania) and depression, accompanied by changes in activity and function, affects about 40 million people worldwide (1, 2). Similar to other common mental illnesses, the current understanding of the etiology of BD is still in its infancy, and the rate of missed diagnoses and misdiagnoses of BD is high (3, 4). These challenges emphasize identifying early risk biomarkers for bipolar disorder (5–7).

Youth identified as being at clinical high-risk for bipolar disorder (CHR-BD) represents a pivotal population for investigating the prodromal phase of the illness (8). These individuals frequently present with subsyndromal mood disturbances, vulnerability to suicidal behaviours, and functional impairments, despite symptoms that are transient or insufficient in severity or duration to meet formal diagnostic thresholds for BD (9, 10).

Non-coding RNA has been widely described as playing a role in brain development, with microRNA (miRNA) being the most extensively studied type. Exosomes are considered a potential source for studying miRNA biomarkers, as they accumulate in greater quantities and are more stable in exosomes, and they are not affected by lysosomal activity (11). In addition, exosomes isolated from blood or urine may be effective diagnostic and prognostic biomarkers for central nervous system diseases (12). In the research of biomarkers based on miRNA, machine learning algorithms can handle high-dimensional datasets and discover patterns and regularities within them. The feature selection process is powerful in identifying the most relevant miRNA features for genomic biomarker discovery through filter, wrapper, or embeded methods (13, 14). Based on the known miRNA expression profiles, they can predict the disease status or other biological characteristics of samples, which is of great significance for early diagnosis research (15).

Research has reported abnormal miRNA expression in patients with BD, and genetic studies have identified *MIR* genes at BD susceptibility loci (16). These findings collectively suggest that miRNAs play a crucial role in the pathogenesis of BD. In recent years, studies have summarized miRNAs related to BD phenotypes including miR-34a, miR-137, miR-499, miR-708, and miR-1908 (16–21). The current research on exosomal miRNA in BD is very limited. When studying exosomal miRNA in post-mortem brain tissue of BD patients, it was found that the level of miR-34a in the cerebellum was elevated, while the level of miR-34a in the prefrontal cortex was decreased (18, 19). Another study based on plasma exosomal samples revealed significant differences in 4 miRNAs between BD patients and healthy individuals (miR-142-3p, miR-484, miR-652-3p, miR-185-5p) (22). Therefore, the change in gene expression influenced by miRNA markers provides a potential mechanism for the regulation of BD phenotype. However, the differential expression of exosomal miRNA has not been reported in the high-risk population of BD.

In this study, we conducted exosomal miRNA sequencing analysis among BD patients, CHR-BD individuals, and healthy individuals. We aimed to carry out group comparisons and model construction based on two assumptions (1): Three distinct groups exhibit different exosomal miRNA expression profiles (2); Exosomal miRNA biomarkers can predict the BD risk status.

## 2 Materials and methods

### 2.1 Participants

Twenty individuals at CHR-BD, 21 patients with established BD-I/II, and 24 healthy controls (HC) aged between 16 and 30 were enrolled (7), matched by age, sex and ethnicity. Three trained psychiatrists performed mental health assessments of all the subjects using the Mini International Neuropsychiatric Interview V. 7.0 the and the Diagnostic and Statistical Manual of Mental Disorders 5th Edition (DSM-5) for the BD diagnosis and other psychiatric comorbidities (23, 24). At Risk for Mania Syndrome (ARMS) in the Bipolar Prodrome Symptom Scale—Full Prospective (BPSS-FP) (25) and the Bipolar At-Risk criteria (BAR criteria) (26) were applied together to define the CHR-BD in this study. Participants who met the DSM-5 diagnosis of BD-I/II were assigned to the patient group with BD, and those who did not meet the DSM-5 diagnosis of BD I/II but met “ARMS” in the BPSS-FP and the BAR criteria were assigned to the CHR-BD group.

The exclusion criteria for all participants were (1): substance abuse disorder (2), significant head injury or a current medical or neurological condition (3), pregnancy, and (4) intellectual disability with an impact on functioning. The exclusion criteria for the BD patient group additionally included past treatment with lithium, which has been proven to play a role regarding the pharmacogenomics and pharmacoepigenomics in BD (27). To study the characteristics of BD and effectively differentiate it from major depressive disorder (MDD), we excluded samples collected during the depressive phase, defined as having a HAMD score of 17 or higher. In addition, the exclusion criteria for HC were (1) personal or familial history of any DSM-5 identified disorder, and (2) past treatment with psychotropic medications.

Social-demographic data regarding age, sex, ethnicity, body mass index (BMI), medical, smoking and alcohol history were collected by a form specifically designed for the study purpose. The affective state (i.e., current depressive and manic symptoms) was assessed by the Hamilton Depression Rating Scale (HAMD) (28), and Young Mania Rating Scale (YMRS) (29). Global functioning was assessed by the Global Assessment of Functioning Scale (GAF) (30).

### 2.2 Sample collection and preparation

Plasma samples were collected from participants. The solution of thermosensitive polymer (PNIPAM-CD63) was mixed evenly with plasma samples and incubated at room temperature for 40 minutes. The mixture was then placed in a water bath at 37°C for 5 minutes, followed by centrifugation at 12000 rpm for 5 minutes at 37°C. The supernatant was removed, and the precipitate containing exosomes was suspended in PBS. The above water bath and centrifugation steps were repeated 3 times to obtain purified exosomes (31).

### 2.3 Exosomal RNA isolation

Samples were processed according to the TRIzol reagent (QIAGEN) instruction manual. RNA concentration was measured by Qubit^®^ 3.0 Fluorometer (Life Technologies, USA). The integrity of RNA was measured by using 1% gel electrophoresis.

### 2.4 Library preparation and small RNA sequencing

Exosomal RNA sequencing was supported by WUHAN MEDBIO CO, LTD. A total amount of 1 μg RNA per sample was used as input material for the RNA sample preparations. Sequencing libraries were generated using NEBNext^®^ Multiplex Small RNA Library Prep Set for Illumina^®^ (NEB, USA) following the manufacturer’s recommendations and index codes were added to attribute sequences to each sample. Briefly, libraries were prepared by ligating different adaptors to the total RNA followed by reverse transcription and PCR amplification and size selection using 6% PolyAcrylamide Gel. Library quality was assessed on the Agilent Bioanalyzer 2100 system. Finally, the qualified libraries were sequenced on the MGISEQ-2000 platform with SE50.

### 2.5 Data pre-processing and analysis of differentially expressed miRNAs

Clean data (clean reads) were obtained by removing reads containing adapter, reads containing ploy-N and reads of low quality from the raw data. At the same time, Q20, Q30 and GC content of the clean data were calculated. All the downstream analyses were based on clean data with high quality. The clean reads were de-redundant using miRDeep2 software, and the collapsed reads were compared with the reference genome (https://www.mirbase.org/). The index of the reference genome was built using Bowtie 1.

The known miRNA sequences of species were obtained in miRBase. The information on known miRNA expression levels in samples and the prediction of novel miRNAs were obtained using miRDeep2. Differential expression analysis of between groups was performed using the DESeq2. DESeq2 normalizes counts using the median ratio method before performing differential expression analysis with a negative binomial distribution. After FDR correction, miRNAs with |Log_2_FC| > 1 and *P* < 0.05 are considered significantly differentially expressed miRNAs (DE miRNAs).

### 2.6 miRNA selection and model building

Using thresholds of |Log2FC| > 1 and unadjusted *P* < 0.01, the study filtered miRNAs with at least moderate differential expression (32, 33). This method also reduces multicollinearity in high-dimensional datasets by excluding miRNAs weakly or not associated with the phenotype, thereby minimizing redundancy among correlated features.

To identify miRNAs capable of predicting BD risk status (three phenotypes: HC, CHR-BD, and BD), machine learning algorithms were incorporated to discover patterns in the expression profiles of candidate miRNAs after filtering. The study uses two separate feature selection methods to identify candidate miRNA, and the cross-consideration between the results is considered as target miRNA (1): Random Forest (RF) (2); eXtreme Gradient Boosting (XGBoost). In each model, the parameters of the training set are adjusted using 10-fold cross-verification. For each feature selection method, we then use the supervised machine learning method to conduct a two-classification (each group is taken as a positive class and the other two groups as a negative class) and create a predictive classification model based on the features selected from the difference study.

The RF model is an ensemble learning method used for classification or regression, which corrects overfitting by building a large number of decision trees during training. We chose the RF model because they are usually robust against overfitting and can learn nonlinear relationships (34). RF feature selection using Boruta aims to identify all relevant variables in classification (35), and then the miRNA combinations selected by Boruta are combined into the random forest model using the randomForest package, with the package’s out-of-bag error reported as an unbiased estimate of the prediction error. We conducted 300 iterations of the random forest normalized permutation importance function to assess attribute importance using the default settings within the Boruta package in R, with a confidence level set at 0.01. This process was repeated 100 times, retaining only those miRNAs selected in at least 10 of these iterations. We then integrated the miRNAs selected by Boruta into a random forest model using the randomForest package. To optimize the model, we used the caret package to find the best number of trees (250) among 100, 250, 500, 750, 1000, 1250, and 1500. Next, we determined the optimal number of variables per tree node to be 1 within the range of 1 to 4. This approach ensured that our model achieved high predictive power while minimizing the risk of overfitting.

The XGBoost method has been very effectively applied to a series of classification problems and has provided insights into biological datasets. However, it takes longer to compute than some other methods due to the tuning of many hyperparameters, and the results may be difficult to interpret. XGBoost sorts features from most to least important. The model is more robust against overfitting. To decide on regularisation parameters, we used a grid search with 10-fold repeated cross-validation, selecting optimal values: (‘nrounds’: 1250, ‘max_depth’: 2, ‘min_child_weight’: 1, ‘gamma’: 0, ‘colsample_bytree’: 0.8, ‘subsample’: 0.75, ‘learning_rate’: 0.025) (36). Once the miRNA is selected, the model is retrained within the same parameter range (37).

Non-parametric Kruskal-Wallis tests were conducted for each feature miRNA to compare expression levels among the three groups, to determine the significance of each miRNA. Then, the *P*-values were adjusted using the Benjamini-Hochberg (BH) multiple testing correction.

The samples were randomly divided into a training set (70%) and a validation set (30%) based on matching age, gender, and group. To compare the classification performance of the models, this study evaluated the classification accuracy of each model for each patient in the validation set. The performance of each feature selection method was compared using the following evaluation indicators: True Positive (TP), False Negative (FN), True Negative (TN), False Positive (FP); Sensitivity, Specificity, Positive Predictive Value, Negative Predictive Value, Correct Classification Rate. Among them: Sensitivity = TP/(TP + FN), Specificity = TN/(TN + FP), Positive Predictive Value = TP/(TP + FP), Negative Predictive Value = TN/(TN + FN), Correct Classification Rate = (TP + TN)/(TP + TN + FP + FN). The pROC package was used for receiver operating characteristic (ROC) analysis.

### 2.7 Enrichment analysis and database validation

**Figure 1:**
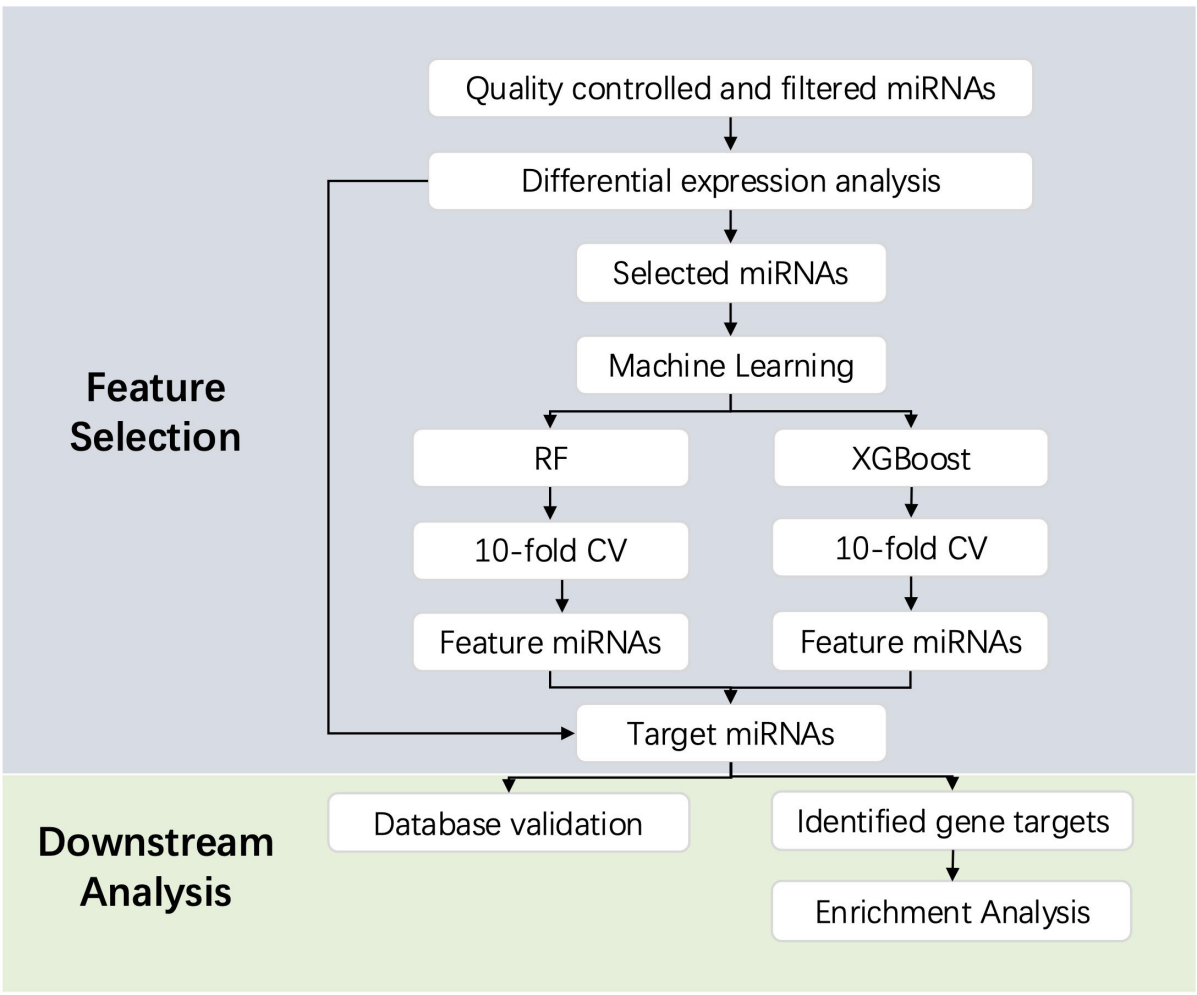
Workflow of the feature selection and downstream analysis in this study.

We then predicted the gene targets for the identified DE miRNAs and target miRNAs on the miRWalk website (http://mirwalk.umm.uni-heidelberg.de/) (38). Based on the database resources of the website, miRNA-target information from at least one of the following databases is selected (1): miRTarBase database (39) (2); TargetScan database (40); (3) miRDB database (41). Target genes are determined based on a score equal to 1. Functional enrichment analysis and pathway enrichment analysis, namely Gene Ontology (GO) analysis and Kyoto Genome Encyclopedia (KEGG) pathway analysis, were performed using the Metascape database (http://www.metascape.org). It is considered statistically significant when Min overlap ≥ 3 and *P* ≤ 0.01 (42). We also used miRNATissueAtlas 2025 (https://www.ccb.uni-saarland.de/tissueatlas2025) to visualize the tissue specifity of the miRNAs (43). The database calculates reads per million mapped (RPM) for 2656 miRNAs, with higher RPM values indicating greater expression levels in a specific organ or tissue. In addition, we searched for associations between miRNAs and neurological diseases in the RNADisease database (http://rnadisease.org/) (44). The workflow is described in **Figure 1**.

## 3 Results

### 3.1 Description of study participants

Demographic data of the participants is shown in **Table 1**. Patients with BD, youth at CHR-BD and healthy controls showed no differences in age, sex, BMI, ethnicity, smoking and alcohol consumption. BD and CHR-BD groups showed higher scores for manic and depressive symptoms and functional impairment than the HC group (*P* < 0.001), and the difference in symptom scale scores between the BD group and the CHR-BD group was not significant (*P* > 0.05). Details on the stratification of the BD group and CHR-BD group are presented in **Supplementary Table 1**.

**Table 1:** Demographic characteristics and symptomatological scales of the three study groups.

Variables	HC (N=24)	CHR-BD (N=20)	BD (N=21)	Total	H/χ2	*P* value
Demographic characteristics						
**Age, mean(SD)**	24.67 (2.26)	23.80 (3.68)	23.86 (3.60)	24.14 (3.17)	1.202	0.548
**Sex, n(%)**					0.815	0.665
Male	7 (29.2)	6 (30.0)	4 (19.0)	17 (26.2)		
Female	17 (70.8)	14 (70.0)	17 (81.0)	48 (73.8)		
**Ethnicity, n(%)**					1.389	0.499
Han	23 (95.8)	18 (90.0)	18 (85.7)	59 (90.8)		
Others	1 (4.2)	2 (10.0)	3 (14.3)	6 (9.2)		
**BMI, mean(SD)**	20.11 (0.20)	21.41 (3.95)	21.21 (3.71)	20.87 (3.41)	1.528	0.466
**Smoke, n(%)**					1.531	0.465
Never	23 (95.8)	17 (85.0)	19 (90.5)	59 (90.8)		
Smoker	1 (4.2)	3 (15.0)	2 (9.5)	6 (9.2)		
**Alcohol, n(%)**					1.085	0.298
Never	13 (54.2)	5 (25.0)	8 (38.1)	26 (40.0)		
Drinker	11 (45.8)	15 (75.0)	13 (61.9)	39 (60.0)		
Symptomatological scales						
**YMRS score, mean(SD)**	0.13 (0.45)	11.10 (15.88)	8.24 (7.84)	6.12 (10.81)	32.464	**<0.001**
**HAMD score, mean(SD)**	1.38 (1.61)	14.32 (7.89)	8.14 (5.66)	7.44 (7.57)	30.93	**<0.001**
**Current GAF, mean(SD)**	91.92 (4.52)	64.74 (15.62)	75.80 (9.81)	78.60 (15.43)	35.425	**<0.001**

HC, Healthy Control; CHR-BD, Clinical High-Risk for Bipolar Disorder; BD, Bipolar Disorder; BMI, Body Mass Index; YMRS, Young Mania Rating Scale; HAMD, Hamilton Depression Rating Scale; GAF, Global Assessment of Functioning Scale.

*P* < 0.05 is considered statistically significant.

### 3.2 Differential expression analysis of exosomal microRNAs

**Figure 2:**
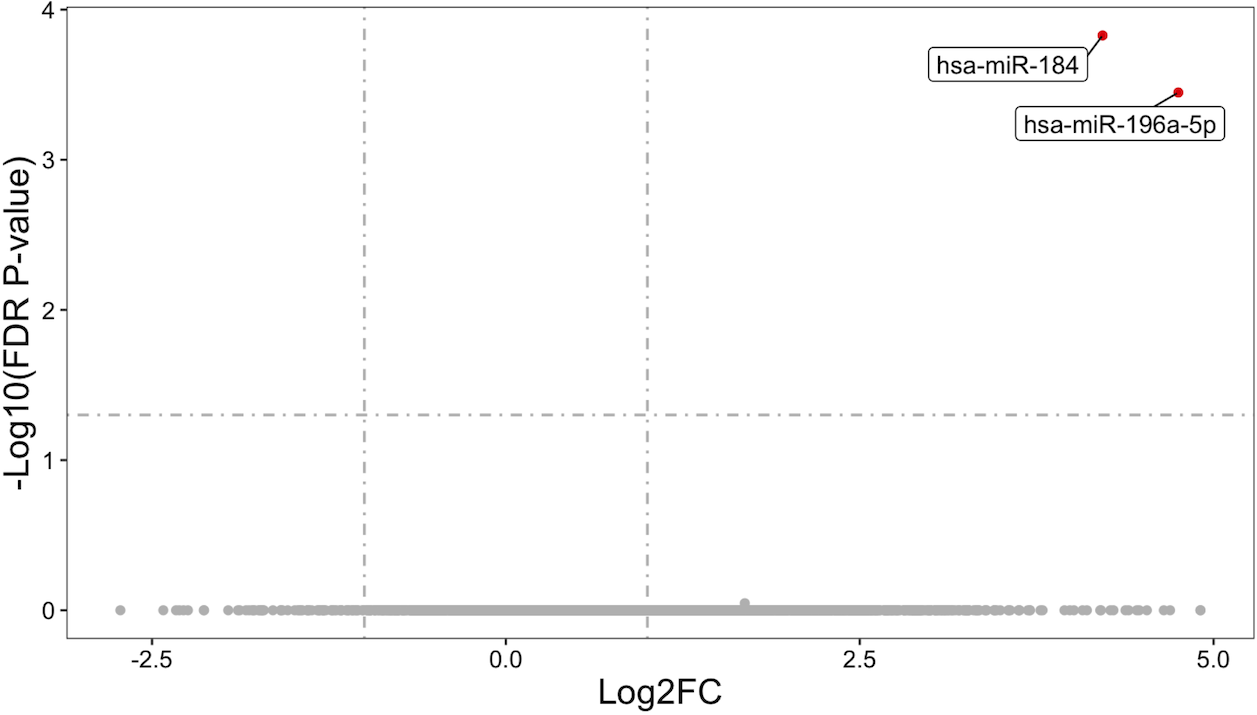
Volcano plot of differentially expressed miRNAs in CHR-BD vs HC. Thresholds: |log2FC| > 1 (vertical lines), FDR-adjusted *P* < 0.05 (horizontal line). Significantly upregulated miRNAs are highlighted.

This study employed DESeq2 for differential expression analysis of miRNAs between groups. After FDR correction, 2 statistically significant DEmiRNAs were found between the CHR-BD group and the HC group: hsa-miR-184 (log_2_FC = 4.22, *P* = 1.49E-04) and hsa-miR-196a-5p (log_2_FC = 4.75, *P* = 3.56E-04) (**Figure 2**). No statistically significant DE miRNAs were identified between the CHR-BD and BD groups, nor between the BD and HC groups after correction (*P* > 0.05, **Supplementary Figure 1**).

The main purpose of this study is to explore the existence of exosomal miRNA biomarkers that can predict BD risk status, namely, the three phenotypes of BD, CHR-BD, and HC. All miRNAs were filtered using thresholds of |log_2_FC| > 1 and unadjusted *P* < 0.01 from intergroup differential expression analyses. This yielded 22 miRNAs with robust effect sizes and moderated statistical significance, which were subsequently advanced to machine learning feature selection.

### 3.3 Machine Learning Feature Selection and predictive performance

**Figure 3:**
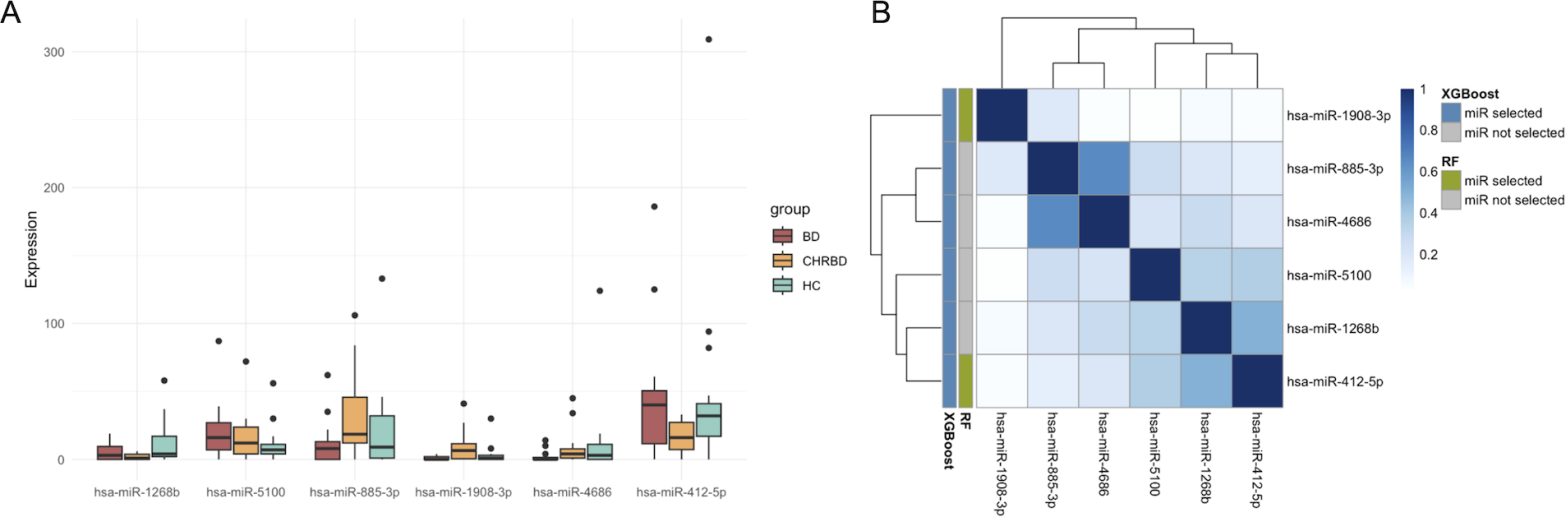
Feature miRNAs identified by machine learning algorithms. **(A)** Average centralization expression value of feature miRNAs; **(B)** Feature selection results and expression correlation matrix. (XGBoost, eXtreme Gradient Boosting; RF, Random Forest).

For the 22 candidate miRNAs, we performed machine learning feature selection using two widely adopted algorithms, RF and XGBoost. A total of 6 feature miRNAs were screened out by the two methods: hsa-miR-1268b, hsa-miR-5100, hsa-miR-885-3p, hsa-miR-1908-3p, hsa-miR-4686, and hsa-miR-412-5p. The expression levels of the six feature miRNAs are detailed in **Figure 3A**. For each feature miRNA, the expression levels of the three study groups were compared using the Kruskal-Wallis test, as detailed in **Table 2**. Both methods jointly selected 2 overlapping miRNAs: hsa-miR-1908-3p and hsa-miR-412-5p (**Figure 3B**).

**Table 2:** Expression levels of six feature miRNAs compared across three study groups using Kruskal-Wallis test.

Feature miRNA	*P*	BH-adjusted *P*
hsa-miR-1908-3p	0.02810198	0.056204
hsa-miR-412-5p	0.011762157	0.056204
hsa-miR-1268b	0.019050246	0.056204
hsa-miR-4686	0.049382394	0.0740736
hsa-miR-5100	0.166966215	0.2003595
hsa-miR-885-3p	0.287990522	0.2879905

BH, Benjamini-Hochberg.

**Figure 4:**
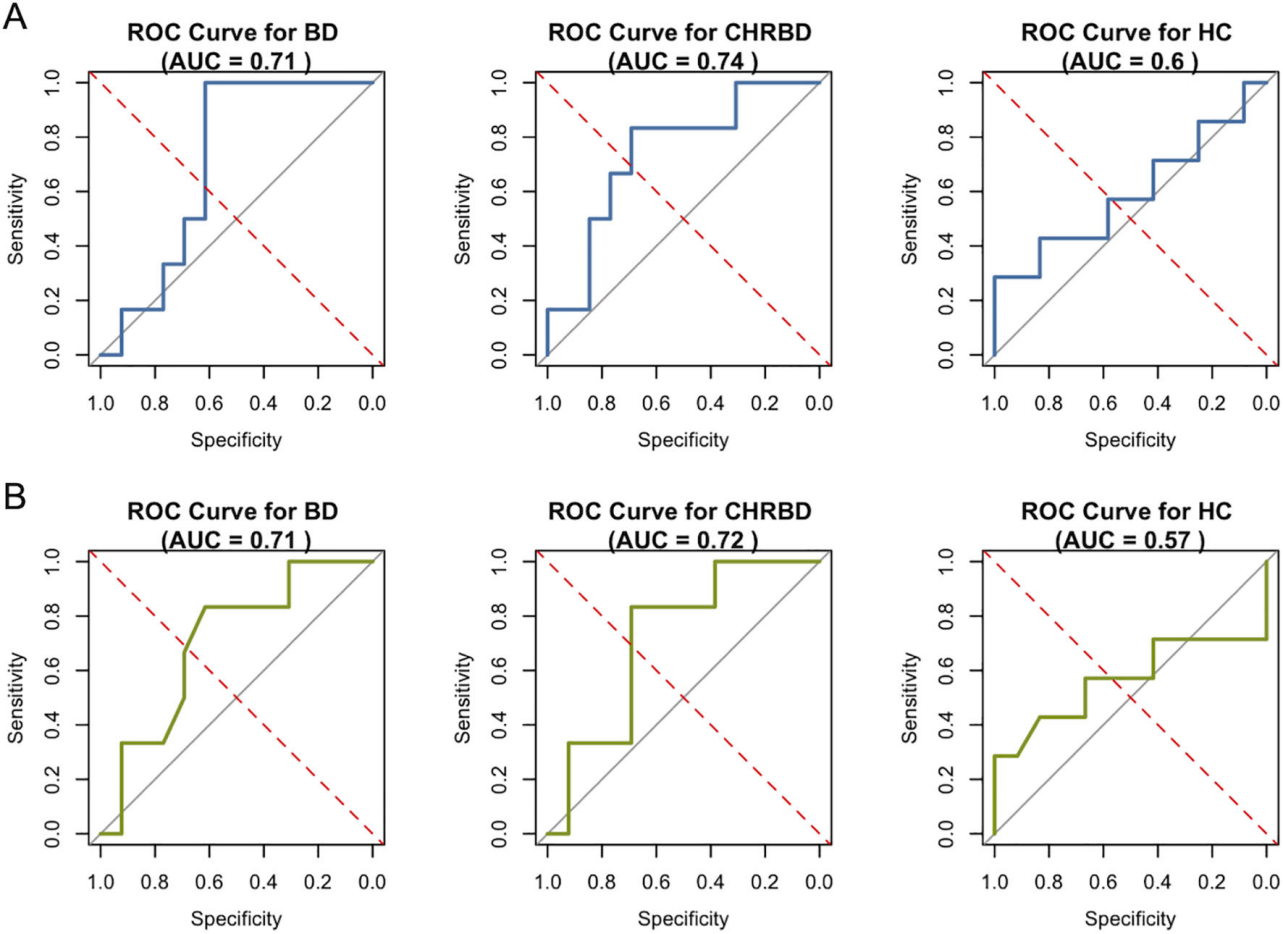
Receiver operating characteristic curves on validation set (n = 19). **(A)** Performance of XGBoost on six feature miRNAs; **(B)** Performance of RF on two feature miRNAs. (XGBoost, eXtreme Gradient Boosting; RF, Random Forest).

As shown in **Table 3**, each feature selection method has different performance on the validation set. XGBoost has a higher area under curve (AUC) when compared to RF (BD group: 0.71 vs 0.71; CHR-BD group: 0.74 vs 0.72; HC group: 0.60 vs 0.57), while the RF model is more stringent, selecting only two miRNAs. The ROC curves of XGBoost and RF on the validation sets are detailed in **Figures 4A, B**, separately.

**Table 3:** Model performance of two classification models (random forest and extreme gradient boosting) on the validation set (n = 19).

	XGBoost (selected six miRNAs)			RF (selected two miRNAs)		
**Evaluation Metrics**	**BD**	**CHR-BD**	**HC**	**BD**	**CHR-BD**	**HC**
**Sensitivity**	0.5	0.5	0.4286	0.8333	0.3333	0.4286
**Specificity**	0.6154	0.8462	0.75	0.6154	0.7692	0.9167
**Positive predictive value**	0.375	0.6	0.5	0.5	0.4	0.75
**Negative predictive value**	0.7273	0.7857	0.6923	0.8889	0.7143	0.7333
**Correct classification rate**	0.5577	0.6731	0.5893	0.7244	0.5513	0.6276
**AUC (95% CI)**	0.71 (0.4647, 0.9456)	0.74 (0.4959, 0.9912)	0.6 (0.2943, 0.8962)	0.71 (0.4518, 0.9584)	0.72 (0.473, 0.9629)	0.57 (0.2299, 0.9011)

XGBoost, eXtreme Gradient Boosting; RF, Random Forest; BD, Bipolar Disorder. CHR-BD, Clinical High-Risk for Bipolar Disorder; HC, Healthy Control; AUC, Area Under Curve; CI, Confidence Interval.

### 3.4 Enrichment analysis and database validation

**Figure 5:**
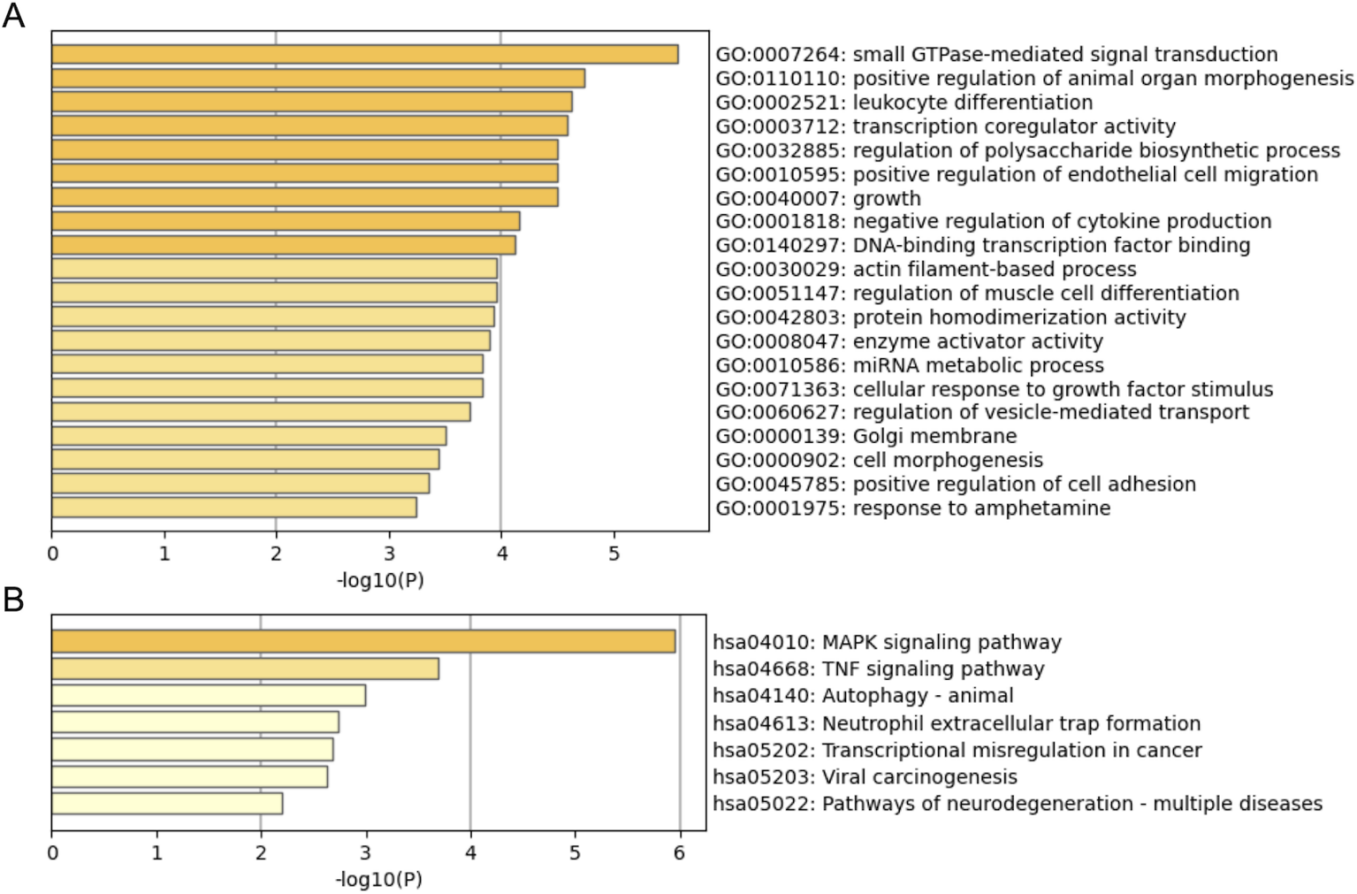
Downstream enrichment analyses of 136 target genes for four miRNAs (hsa-miR-184, hsa-miR-196a-5p, hsa-miR-1908-3p and hsa-miR-412-5p). **(A)** GO functional enrichment results; **(B)** KEGG pathway enrichment results.

To study the role of miRNA in BD risk, the 2 differentially expressed miRNAs (hsa-miR-184 and hsa-miR-196a-5p) obtained from the comparison between the CHR-BD and HC groups, as well as the 2 target miRNAs (hsa-miR-1908-3p and hsa-miR-412-5p) selected simultaneously by two models, target gene prediction was conducted using the miRWalk website, identifying a total of 136 unique target genes (**Supplementary Table 2**). Subsequently, GO and KEGG enrichment analyses were conducted on the Metascape database to further understand the biological functions and pathways associated with these target genes (**Figures 5A, B**). The miRNA specific expression across normal human organs and brain tissues are shown in **Supplementary Figure 2**.

When the training sample is small, the feature selection processes may be unstable. To address this, for the four target miRNAs (hsa-miR-184, hsa-miR-196a-5p, hsa-miR-190 8-3p and hsa-miR-412-5p), we investigated the relationship between these miRNAs and neurological diseases using the RNADisease database (**Table 4**). All four miRNAs were found to be associated with neurodegenerative diseases: Parkinson’s disease (4/4), Fronto-temoral dementia (3/4), and Alzheimer’s disease (2/4). In addition, hsa-miR-184 is also related to autism spectrum disorder.

**Table 4:** Summary of evidence on four target miRNAs and neurological diseases from the RNADisease database.

Findings in this article			Evidence in the literature				
	miRNA	CHR-BD group	Trait	Disease group	Sample	PMID	Score (0-1)
DE miRNAs between CHR-BD and HC group	hsa-miR-184	Up-regulated	Autism Spectrum Disorder			18203756	1.00
			Alzheimer Disease	Up-regulated	Brain	20936480	0.97
				Differential express	Brain	23822153	
				Differential express	Blood, Cerebrospinal fluid	24797360	
				Differential express	Brain	28127595	
				Down-regulated	Temporal cortex	28137310	
			Parkinson Disease	Up-regulated	Fibroblasts	29986767	0.64
						33748099	
			Mild Traumatic Brain Injury			32024417	0.33
			Frontotemporal Dementia			33800495	0.33
	hsa-miR-196a-5p	Up-regulated	Parkinson Disease	Up-regulated	iPSCs	29986767	0.33
			Frontotemporal Dementia			33800495	0.33
Overlapped feature miRNAs in RF and XGBoost	hsa-miR-1908-3p	Up-regulated	Parkinson Disease	Up-regulated	Neuron	29986767	0.33
			Frontotemporal Dementia			33800495	0.33
	hsa-miR-412-5p	Down-regulated	Alzheimer Disease	Differential express	Brain	28127595	0.33
				Down-regulated	Temporal cortex	28137310	
			Parkinson Disease	Up-regulated	Neuron	29986767	0.33

CHR-BD, Clinical High-Risk for Bipolar Disorder; DE miRNAs, significantly Differentially Expressed miRNAs; iPSCs, induced Pluripotent Stem Cells.

## 4 Discussion

In recent years, molecular biological research on BD has indicated that miRNAs may play a crucial role in the disease’s pathogenesis (17, 19, 21, 45). To our knowledge, this study pioneers the study of exosomal miRNA sequencing in individuals at clinical high risk for BD. We introduced two feature selection models to explore the potential of intergroup differential miRNAs as early risk markers for BD, advancing our understanding of BD’s underlying mechanisms and potentially enhancing early detection and intervention strategies.

Our differential analysis highlights that, compared to the HC group, two miRNAs (hsa-miR-184 and hsa-miR-196a-5p) are significantly upregulated in the CHR-BD group. Interestingly, no miRNAs showed statistically significant differences between the CHR-BD and BD groups, nor between the BD and HC groups. hsa-miR-184 is a highly conserved, single-copy miRNA that exhibits specific expression patterns in the adult brain, particularly within the subependymal zone and the dentate gyrus of the hippocampus. This miRNA plays a crucial role in neurodevelopment and synaptic plasticity. Overexpression of hsa-miR-184 promotes the proliferation of neural stem cells and significantly reduces neuronal differentiation (46). A previous study has shown that hsa-miR-184 is significantly downregulated in patients with late-onset depression, and the knockout of its homologous gene in the fruit fly can lead to reduced motor activity and impaired memory function, suggesting that its downregulation may be related to the neurobehavioral abnormalities associated with late-onset depression (47). Similar to the findings of this study, a previous study on post-mortem brain ACC tissue indicated that the expression level of hsa-miR-184 in MDD patients is lower than that in healthy controls, while there is no significant difference in the expression level in BD patients compared to healthy controls (16). Our study found an upregulation of hsa-miR-184 in the CHR-BD group, and the expression in the BD group was also non-significantly higher than that in the HC group (log_2_FC = 1.503, unadjusted *P* = 0.046, data not shown). Considering the above, hsa-miR-184 appears to play a role in the mechanisms of mood disorders. However, further investigation is needed to understand the differences in related molecular mechanisms between BD and MDD, and future studies could explore hsa-miR-184 as a potential biomarker in mood disorders. hsa-miR-196a-5p is involved in the development and progression of tumours and has been extensively studied as a biomarker for various cancers (48). In addition, it has been reported to have the potential as a disease biomarker in pediatric epilepsy (49) and Huntington’s disease (50). In a study on diagnostic markers for pediatric epilepsy, hsa-miR-196a-5p was identified as a key diagnostic marker in plasma extracellular vesicles (AUC = 0.840), and its logistic regression model with other three miRNAs has good diagnostic potential (AUC = 0.940). The related target genes were significantly enriched in the PI3K-Akt and MAPK signalling pathways, suggesting that they may participate in the occurrence of epilepsy by regulating neuronal excitability and cell proliferation (49). It is noteworthy that the above two signalling pathways have also been widely reported to be related to synaptic plasticity, neuroimmunology, and lithium treatment response in BD (51–53). In this study, the upregulation of hsa-miR-196a-5p may lead to an imbalance in neuronal homeostasis in clinical high-risk populations by inhibiting key molecules in related pathways, thereby increasing the risk of transitioning to (hypo)manic or cyclothymic episodes. It is worth noting that no miRNA was significantly differentially expressed between the BD (13/21 at euthymic episode) and HC groups, which may suggest a dynamic change in miRNA levels associated with mood symptoms. Future studies employing larger, mood-stratified cohorts should aim to disentangle pathology-specific signals from state-dependent fluctuations.

Through machine learning feature selection, our analysis identified two miRNAs (hsa-miR-1908-3p and hsa-miR-412-5p) that may be associated with BD risk. Notably, these two miRNAs are also selected by the XGBoost model. miR-1908 was previously identified as one of the three most promising BD diagnostic biomarkers in a genome-wide association study (GWAS) including 9747 BD patients and 14278 controls, with 2.3 million SNPs and 700 miRNAs (20). The top SNP associated with miR-1908 was rs174575 (20), which also demonstrated genome-wide significance (*P* = 7.18E-10) in a more recent GWAS study involving over 40000 BD cases (54). The *MiR-1908* gene is located in the first intron of the fatty acid desaturase 1 (FADS1) gene on chromosome 11, and the target gene network regulated by it shows significant enrichment in neuron projection and nervous system development. The results of RT-qPCR studies using peripheral blood samples indicate that compared with remission status (defined as HDMD < 8), BD patients have lower expression levels of miR-1908 (*P* = 0.004) during depressive episodes, suggesting the potential of the this miRNAs as biomarkers for BD disease status (45). Our findings strengthen the evidence supporting the association between hsa-miR-1908-3p and BD symptoms. Future studies should further investigate this relationship in different emotional symptom subgroups of BD risk phenotypes. Research on hsa-miR-412-5p is limited, but its potential value in the early identification of amyotrophic lateral sclerosis (ALS) has been preliminarily confirmed. It may function by regulating the PI3K-Akt pathway and targeting genes like *BCL2* and *OPTN*, thereby contributing to neurodegenerative changes in ALS (55). The above pathways and genes have been identified in BD (56–59). However, the pathological mechanisms of its involvement in BD high-risk courses warrant future investigations.

We explored the association between the above four target miRNAs with neuropsychiatric disease phenotypes through searching the RNADisease database, which revealed links between all four miRNAs and neurodegenerative disorders. These miRNAs may regulate neurodevelopmental processes and neurodegenerative changes through mechanisms like neuroinflammation, abnormal synaptic plasticity, or protein homeostasis imbalance (60). Given the unique diagnostic potential of circulating miRNAs in central nervous system disorders (61), future studies should prioritize validating their value in identifying BD risk.

In our model validation, the XGBoost algorithm outperformed RF with higher AUC values, likely due to its ability to handle complex, non-linear relationships in miRNA data (37). Despite this, miRNA-based models are still insufficient in distinguishing BD from healthy controls at an individual level, possibly due to BD’s multifactorial nature where miRNA expression captures only part of the biological heterogeneity. Similar to differential analyses, these models suggest that exosomal miRNAs are more effective for identifying CHR-BD populations than for delineating BD characteristics. The subtle (albeit non-significant) higher scores in HAMD and YMRS observed in CHR-BD groups compared to BD groups might hint at symptom-linked miRNA dynamics, suggesting that the targeted miRNA profiles may align more closely with dynamic, disease risk-related changes. Future longitudinal studies with larger sample sizes that follow up on BD conversion outcomes are essential in high-risk studies. As mood state may influence inflammatory (16) and miRNA markers (45), these studies should focus on subgroup analyses based on dynamic emotional states (e.g., euthymia, depression, hypomania, mixed) to clarify whether miRNA patterns reflect transient mood episodes or stable risk signatures (62). Interating clinical characteristics with multi-omics data is essential to uncover BD-specific early regulatory networks.

In the present study, GO analyses revealed critical biological processes, including neuroinflammation, synaptic plasticity dysregulation, and epigenetic remodelling, that may underpin early-stage BD pathophysiology. Supporting this, KEGG pathway analysis highlighted the MAPK and TNF signalling pathways, both of which are mechanistically intertwined with neuroinflammatory cascades, stress adaptation, and synaptic integrity (63, 64). These pathways are particularly salient in BD, where chronic stress and immune dysregulation are hypothesized to drive neuronal vulnerability. For instance, MAPK signalling not only modulates inflammatory responses but also influences synaptic plasticity and neurogenesis, bridging molecular dysfunction with behavioural phenotypes observed in BD (65, 66). Similarly, TNF-α, a key mediator of neuroinflammation, has been implicated in mood dysregulation and neuronal apoptosis, offering a plausible link to BD progression (65–67). These findings align with existing models of BD pathogenesis (68). Future efforts would clarify whether these pathways represent actionable targets for halting or reversing disease progression in BD at-risk individuals. The specific expression results reveal distinct tissue-specific patterns for the identified miRNAs, with hsa-miR-184 and hsa-miR-412-5p notably enriched in brain regions, highlighting their potential functional importance in brain-related processes.

## 5 Limitation

This study has several limitations. Firstly, the sample size was relatively small, which limited the representativeness of the population and the statistical effectiveness of the results. Secondly, we recruited all participants aged 16 to 30, including BD patients. This age range could lead to an overrepresentation of early- or mid-onset patients, as late-onset BD patients may be underrepresented or excluded (69). However, our sample was selected based on a good match to the CHR-BD group, and early-onset patients may better reflect the genetic characteristics of BD. Thirdly, although we used BARS standards and ARMS in BPSS-FP to define the CHR-BD population, further prognostic verification is necessary. The heterogeneity within the CHR-BD population necessitates future cohort studies to identify subgroups at higher risk of converting to BD. Fourthly, we did not conduct a subgroup analysis based on different emotional states or BD diagnostic subtypes due to the limited sample size. Future studies with larger sample size should incorporate various mood states to clarify whether miRNA patterns reflect transient mood episodes or stable risk signatures. Lastly, while our study utilized peripheral blood exosomal miRNAs as a practical proxy for brain-derived signals, cross-tissue variability in miRNA expression remains a critical consideration. However, blood-based biomarkers are preferable for early BD detection due to the inaccessibility of brain tissue in clinical settings, future work should integrate cell/tissue-specific analyses with post-mortem brain samples, cerebrospinal fluid samples, or animal models to validate the functional relevance of these miRNAs to neuropathology.

## 6 Conclusion

This study investigated exosomal miRNA in the CHR-BD population, revealing four miRNAs with intergroup differences linked to BD risk. These miRNAs are implicated in neuroimmunity and neuronal plasticity, with literature supporting their link to neuropsychiatric diseases, especially mood disorders. Therefore, these miRNAs and their related targets and pathways can be considered potential biomarkers for early BD recognition. Our predictive model developed for exosomal miRNAs represents a novel investigation in identifying clinical risk phenotypes in BD. While the current miRNA panel remains insufficient to fully unlock the clinical utility of risk model for early BD prediction, future cohort studies involving larger samples and integrating multidimensional biomarkers are poised to bridge this gap. Such efforts promise to enhance predictive accuracy and facilitate proactive, personalized strategies in BD early identification and prevention.

## Data Availability

The miRNA-Seq data in the study are deposited in the NCBI SRA repository with accession number PRJNA1264007 and are accessible at the following link: https://www.ncbi.nlm.nih.gov/bioproject/PRJNA1264007.

## Supplementary material

Supplementary Figure 1
**(A)** Volcano plot of differentially expressed miRNAs in CHR-BD vs BD; **(B)** Volcano plot of differentially expressed miRNAs in BD vs HC. Thresholds: |log2FC| > 1 (vertical lines), FDR-adjusted *P* < 0.05 (horizontal line).

Supplementary Figure 2 Organ and brain tissue specificity of four miRNAs (hsa-miR-184, hsa-miR-196a-5p, hsa-miR-1908-3p and hsa-miR-412-5p) associated with clinical risk of bipolar disorder. **(A)** Organ specificity; **(B)** Brain tissue specificity. (rpmm: reads per million mapped).
