# Supplementary figures and images for: Exosomal microRNA signatures in youth at clinical high risk for bipolar disorder
Source: Front Psychiatry. 2025 May 20;16:1589374. doi: 10.3389/fpsyt.2025.1589374 (PMC12131863; doi:10.3389/fpsyt.2025.1589374)

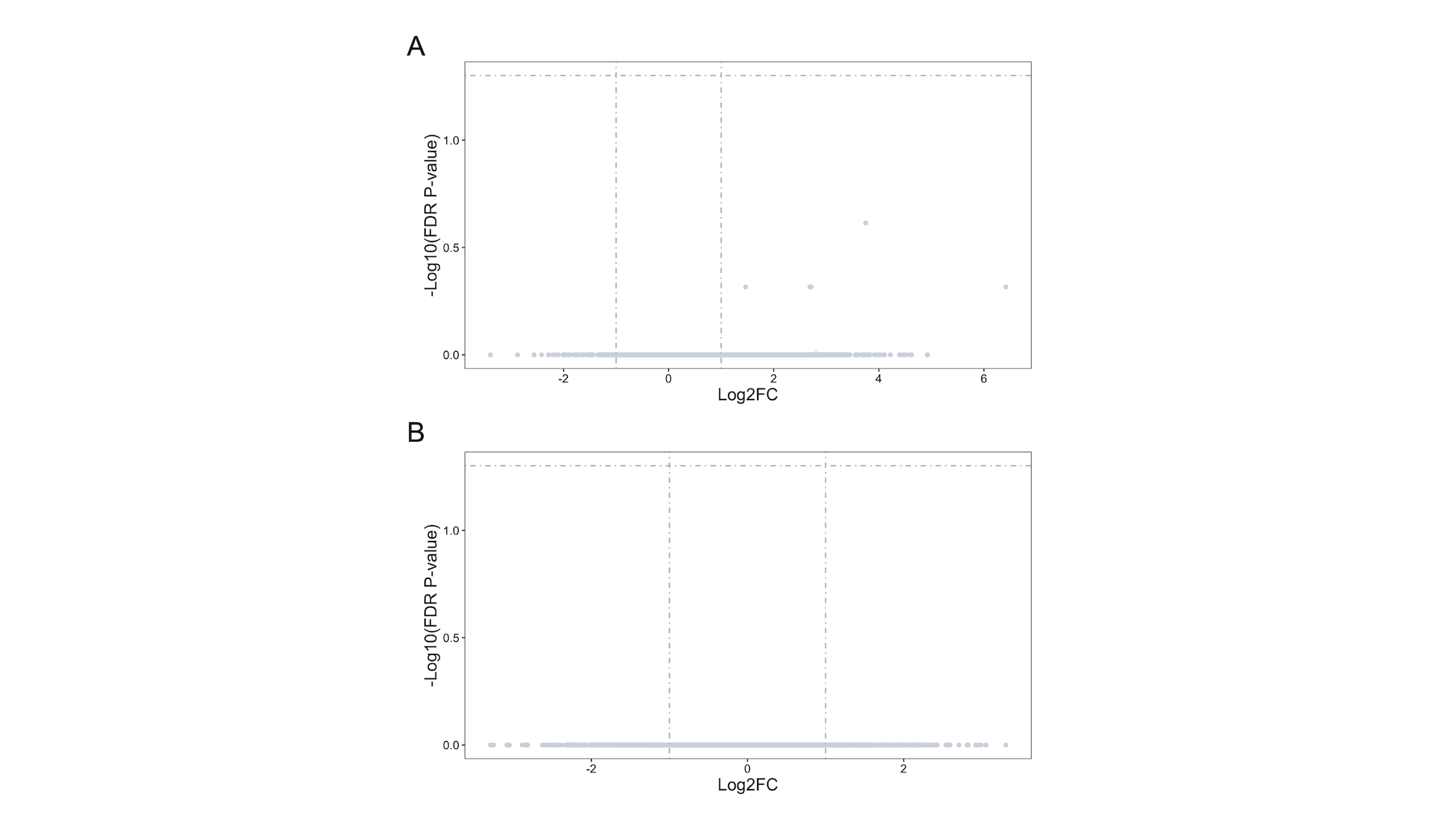

Supplement: Supplementary Figure 1 — (A) Volcano plot of differentially expressed miRNAs in CHR-BD vs BD; (B) Volcano plot of differentially expressed miRNAs in BD vs HC. Thresholds: |log2FC| > 1 (vertical lines), FDR-adjusted P < 0.05 (horizontal line). [file Image1.tiff]

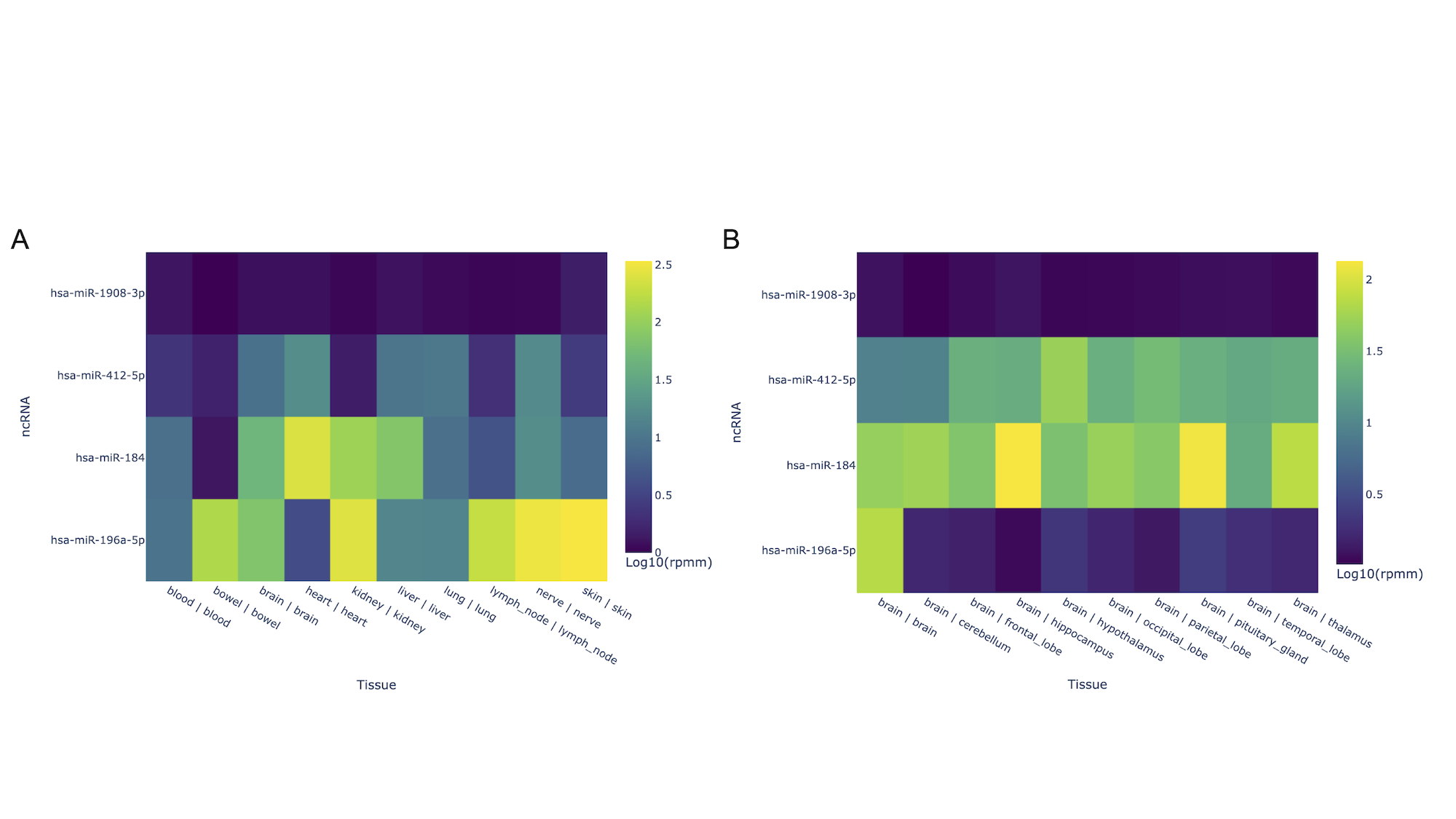

Supplement: Supplementary Figure 2 — Organ and brain tissue specificity of four miRNAs (hsa-miR-184, hsa-miR-196a-5p, hsa-miR-1908-3p and hsa-miR-412-5p) associated with clinical risk of bipolar disorder. (A) Organ specificity; (B) Brain tissue specificity. (rpmm: reads per million mapped). [file Image2.tiff]
